# CASCADE: A Community-Engaged Action Model for Generating Rapid, Patient-Engaged Decisions in Clinical Research

**DOI:** 10.21203/rs.3.rs-4790564/v1

**Published:** 2024-08-27

**Authors:** Bridgette L. Kelleher

**Affiliations:** Purdue University

**Keywords:** Community-based participatory research, decision making, clinical trials, patient engagement, CASCADE, Delphi panel, Project WellCAST, patient acceptability

## Abstract

**Background:**

Integrating patient and community input is essential to the relevance and impact of patient-focused research. However, specific techniques for generating patient and community-informed research decisions remain limited. Here, we describes a novel CASCADE method (Community-Engaged Approach for Scientific Collaborations and Decisions) that was developed and implemented to make actionable, patient-centered research decisions during a federally funded clinical trial.

**Methods:**

The CASCADE approach includes 7 key pillars: (1) identifying a shared, specific, and actionable goal; (2) centering community input; (3) integrating both pre-registered statistical analyses and exploratory “quests”; (4) fixed-pace scheduling, supported by technology; (5) minimizing opportunities for cognitive biases typical to group decision making; (6) centering diversity experiences and perspectives, including those of individual patients; (7) making decisions that are community-relevant, rigorous, and feasible. Here, we implemented these pillars within a three-day CASCADE panel, attended by diverse members of a research project team that included community interest-holders. The goal of our panel was to identify ways to improve an algorithm for matching patients to specific types of telehealth programs within an active, federally funded clinical trial.

**Results:**

The CASCADE panel was attended by 27 participants, including 5 community interest-holders. Data reviewed to generate hypotheses and make decisions included (1) pre-registered statistical analyses, (2) results of 12 “quests” that were launched during the panel to answer specific panelist questions via exploratory analyses or literature review, (3) qualitative and quantitative patient input, and (4) team member input, including by staff who represented the target patient population for the clinical trial. Panel procedures resulted in the generation of 18 initial and 12 final hypotheses, which were translated to 19 decisional changes.

**Conclusions:**

The CASCADE approach was an effective procedure for rapidly, efficiently making patient-centered decisions during an ongoing, federally funded clinical trial. Opportunities for further development will include exploring best-practice structural procedures, enhancing greater opportunities for pre-panel input by community interest-holders, and determining how to best standardize CASCADE outputs.

**Trial registration:**

The CASCADE procedure was developed in the context of NCT05999448.

## BACKGROUND

Integrating patient and community input into decision making is essential to the relevance and impact of patient-focused science (1). However, specific techniques for community-informed decision making remain limited. Practical, *in vivo* community engagement techniques are particularly lacking, with most guidelines focusing on the broad steps to community-engaged research rather than the strategies that researchers can use to involve patients and communities in real-time. The present manuscript describes a novel CASCADE method (Community-Engaged Approach for Scientific Collaborations and Decisions) that we recently developed and implemented to make actionable, patient-centered research decisions during a federally funded clinical trial. We first describe the justification and empirical motivation for developing CASCADE, including how the approach differs from other community-centered and consensus-generating methods. We then describe the technical protocol for implementing CASCADE, including results from an inaugural panel implemented during an active clinical trial. We conclude by discussing key takeaways from CASCADE implementation and next steps for methodological development and validation.

### Methods for Summarizing Consensus Across Patients and Community-Members

The voice of the patient is central to any clinical research endeavor. Patient engagement in research has been systematically defined as *“the active, meaningful, and collaborative interaction between patients and researchers across all stages of the research process, where research decision making is guided by patients’ contributions as partners, recognizing their specific experiences, values, and expertise.”* (3, p. 682). A variety of methods have been used to engage patients in healthcare and research contexts (3), including involvement of a patient advisory councils (4), patient-led provider training (5), and co-designing research programs (6); large-scale meta-analyses have supported the efficacy of such programs on health outcomes, particularly when communities are directly involved in health-related interventions (7). More passive methods for considering patient experiences are also common, such as the evaluation of patient behavior (e.g. attrition, compliance) or patient-reported surveys to assess acceptability of healthcare interventions (8). Increasingly, patient communities are self-organizing to impact and control research decisions, including by developing research resources such as registries (9, 10) and, in some cases, directly financing and co-creating research relevant to their community (11).

Patient-engaged research can be conceptualized as a type of participatory research, which broadly aims to engage potential users of research into the design and application of the research itself (1). Participatory methods, including community-based participation research (CBPR) methods, have historical roots in Kurt Lewin’s *action research* movement, which aimed to engage minority participants in the translation of complex social issues to social action through a sequence of fact finding, taking action, and evaluating impact (12). At present, CBPR is generally characterized as a collaborative research approach that integrates equitable input from community, organizational, and research interest-holders (13, 14). Israel and colleagues (13) have summarized key tenants of CBPR, including many principles relevant to patient in research-related decisions. However, the current status quo is that few patient-focused endeavors fully align with these CBPR tenants. One particular challenge to CBPR is the often-unclear process for how to best synthesize patient perspectives into actionable outputs (13). Rigorous qualitative methods that are often used in CBPR, such as focus groups and intensive interviews (15, 16), are also often time-consuming and resource-intensive, posing challenges for rapid decision-making contexts. Methods for more generally engaging with community advisory groups are not well-standardized, and there is little accountability for researchers to integrate and act on community input in these contexts. Thus, additional frameworks are needed to translate CBPR into acute, patient-engaged decision-making contexts.

### Methods for Building Consensus across Experts and Lay Experts

A variety of methods have been developed to generate consensus or agreement in medical research(17, 18) and offer a starting point for building a model for how to generally build consensus on patient-relevant topics. For example, the Delphi method(19) is a highly popular, systematic process for making complex decisions by iteratively integrating expert input toward consensus across multiple rounds of anonymous expert feedback. However, in contrast to CBPR principles, this structure assumes that group-based decisions provide greater value and stability than individual input (20), and that discussion weakens decision-making by introducing biases and uneven input (19–21). Other models for consensus and decision-making have – such as the RAND/UCLA Appropriateness Method(22) and consensus development conferences(23) - include more discussion and input from lay experts (17, 23). However, similar to Delphi panels, these methods focus on summarizing expert opinion and require extensive resources to execute, limiting utility for CBPR.

A fourth common model for consensus - nominal group technique(24, 25) – incorporates several elements that align with the goals of CBPR. Similar to other consensus models, nominal group technique involves a multi-step phase that includes structured presentation of input, feedback to the group, discussion, and voting to rank-order outputs. A key distinction of this method is that prior to this process, group members engage in is “nominal” activities such as independent, written responses to pre-determined prompts, with the goal of minimizing the biases and power imbalances and enhancing creative outputs (25). Technique developers Van de Ven and Delbeco (1971) explicitly note that providing time for individual reflection and input prior to group discussion may “encourage the generation of minority opinions and ideas” and “alleviate… covert political group dynamics which are difficult to develop when writing” aligning with CBPR principles. Although nominal group techniques are typically applied to gather consensus among experts, the approach is increasingly used to identify consensus amongst patients (26, 27), supporting nominal group technique as a potential starting point for integrating patients in more rapid decision-making contexts.

A common criticism of consensus-driven methods, including nominal group technique, is the potential to dilute novel ideas and focus policy and decisions at the level of a “lowest common denominator” (23). Indeed, a variety of cognitive biases have been described to impact decision making, particularly in group contexts, and are purported to impact patient outcomes (28). To minimize the potential impact of such biases in consensus generation, Bhandari and colleagues (29) generated a guide to identifying and reducing specific cognitive biases that can compromise group-based decision making. For example, they suggest that iterative rounds of discussion with descriptive feedback and minimize potential the *false consensus effect* (30), a tendency to over-estimate the degree to which others agree with one’s own opinion. Their guidelines provide a useful metric for considering how methodological decisions impact the rigor of consensus-based decisions, particularly when designing new approaches to integrating patient voices into consensus-based research.

## The Present Study

Although a variety of CBPR and consensus-based decision-making approaches have been developed, the field lacks tangible guidelines for how to best integrate patients and community-interest holder input into real-time clinical research decision-making. As part of our current NIH-funded clinical trial, we addressed this gap by developing a collaborative, community-informed approach for integrating interest-holder input with other sources of data to make tangible decisions about project design. This decision-making process centered on a core scientific decision within the study: the development of a precision health algorithm that determined which forms of clinical support were assigned to which trial participant. The present manuscript introduces the CASCADE method (Community-Engaged Approach for Scientific Collaborations and Decisions) developed for this task. Here, we describe the guiding principles and technical protocol for implementing CASCADE, using our inaugural panel as an example. We conclude by discussing “lessons learned” from our first CASCADE panel, including next steps in the development and application of this method.

## METHODS

### Guiding Pillars of CASCADE

The purpose of the CASCADE method is to rapidly synthesize multiple sources of data with community and scientific input to make acute research decisions. CASCADE was informed by action research (31), best-practice CBPR approaches (14), nominal techniques (24), and best practices for minimizing cognitive biases in consensus approaches (29). CASCADE included seven guiding pillars:

#### Pillar #1: Identify a shared, specific, actionable goal.

CASCADE is designed to answer a specific, pre-defined question. In this way, CASCADE has structural similarities with initial action research approaches that aimed to distil complex issues into actionable progress (12, 31). In cases in which a clear goal is not fixed in advance, a variety of CBPR principles and techniques can be used to facilitate shared decision making around research questions and agendas (13, 16, 32, 33). Similarly, a number of methods have been developed to ensure goals are well-described; for example, SMART goals are created to be specific, measurable, achievable, realistic, and time-based (34).

#### Pillar #2: Center community input.

*Direct Community Input* was represented within this group by our “peer coaches,” caregivers of children and adults with rare disorders (the target population for the trial) who were paid part-time staff on the project. Within the broader project, peer coaches help design and plan elements of the project, implement a portion of support programs, support recruitment and community engagement, and assist with data interpretation and dissemination. Given we were discussing confidential information, having paid, human subjects-certified staff on our team who could provide input and hands-on perspective was central to the success of CASCADE. Because peer coaches interacted directly with participants about their experiences in the trial, they were also able to offer anecdotal information about their observations and perceptions about patient experiences.

*Indirect Participant Input* was represented through both patient-reported and behavioral data, per general field standards (8). Patient-reported data included quantitative survey responses and qualitative responses to open-ended questions. To adapt for a rapid-paced discussion within 2 weeks of data collection, we summarized qualitative input in three ways. First, two peer coaches with read all qualitative responses and provided written, item-by-item summaries of their contents prior to the meeting; during the meeting, they served as designated “representatives” of the data and continuously reflected on what they had studied as applicable to the current context. Second, we used artificial intelligence (AI) through ChatGPT 4.0 (35) to similarly summarize item-by-item responses; inputs included the item question and all de-identified data (see Pillar #4). We conceptualized AI-generated data as a descriptive tool rather than a proxy of gold-standard qualitative coding. Third, a student researcher with prior qualitative coding experience also summarized the data. Thus, we triangulated peer coach, AI, and qualitative expert summaries to ensure accuracy, completeness, and representativeness of the participants’ data. We also evaluated implied patient experiences by integrating observational proxies of participant outcomes such as drop-out, session completion, and homework completion (8).

#### Pillar #3: Integrate both pre-registered statistical analysis and exploratory “quests.”

A primary focus of CASCADE was on pre-registered statistical analyses. The benefits of statistical pre-registration have been well described in the literature (36), building on a rich history of protocol registration that is common, and often required, for medical and clinical research (37). Pre-registration is important to reducing potential biases, increasing transparency, and minimizing what has been described as “researcher degrees of freedom,” (38) subtle ways in which researchers’ design and analysis decisions can intentionally or unintentionally bias results (36). In the context of CASCADE, preregistration is particularly important to distinguish planned analyses, which we implemented to test or core hypotheses, from exploratory analyses that functioned to help generate hypotheses for the next wave of data collection.

A second major focus of CASCADE was to develop and evaluate novel hypotheses. Here, we incorporated the idea of “Quests,” defined as rapid, targeted data analysis or literature review executed with the purpose of evaluating evaluate the relative strength of a new hypothesis. These exploratory analyses were not designed to produce generalizable knowledge about the target population, but rather to consider the strengths and weaknesses of proposed hypotheses and action items. Quests were designed to be limited in scope, capable of being completed in 1 hour or less in between panel meetings, and directly related to specific hypotheses. Quests were completed by project staff, including biostatisticians and student or postdoctoral trainees, and were verified for accuracy after the panel, prior to final implementation.

#### Pillar #4: “Peel the onion” at a fixed pace, with support from technology.

The CASCADE model is focused on efficient decision-making, which comes at an obvious and expected cost to discussion depth. We conceptualized our task during CASCADE as peeling an onion, with the understanding that we could only get to so many layers in a given period of time. As such, the agenda for each day was fixed in advance, with minimal deviation, and it was acknowledged that we would not be able to fully explore all possibilities during the project. To maintain this pace, we prepared many of the core documents ahead of the meeting, including statistical analyses, and leveraged pre-meeting surveys to solicit panelist input in advance (24).

**Figure 1:**
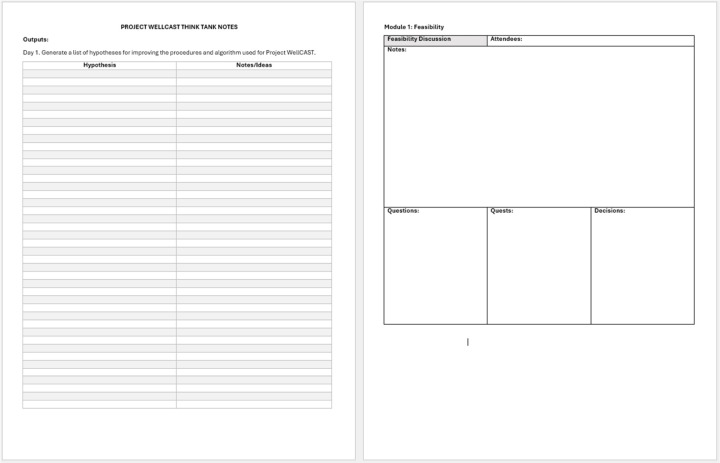
Sample shared note-taking document used to document CASCADE in real time.

We also selectively leveraged technology to support both clerical and data synthesis tasks. Clerically, we relied on a shared note-taking document on Google Docs (39), accessible to all panelists, that documented (1) key hypotheses generated during the meeting, (2) details of each segment of discussion, along with questions, planned quests (Pillar #3), and decisions, and (3) documentation of all project decisions, including how we satisfied our core decisional criteria (Pillar #7); the shell for this document is displayed in Fig. 1. We also leveraged Zoom’s “record meeting” function to save record of the meeting, used for later verification of discussion, and used the chat feature to supplement live dialogue during the meeting.

To support data synthesis, we also used ChatGPT (35) to summarize – but not thematically analyze – both participant and panelist input. ChatGPT has been previously validated to accurately extract concrete and descriptive themes from qualitative data, however its capacity to conduct thematic analyses and detect nuanced patterns is more limited (40). Within CASCADE, we used ChatGPT with these constraints in mind by (1) requesting item-by-item synthesis, anchored to a very specific item question, (2) never uploading sensitive, personal, clinical, or identifiable data, (3) cross-validating ChatGPT with other analysis methods, particularly when summarizing participant input (Pillar #2). In any publications using ChatGPT-derived summaries, we plan to make detailed methods, including prompts, available via osf.io.

#### Pillar #5: Intentionally minimize opportunities for cognitive biases.

Consistent with recommendations by Bhandari and colleagues (29), we sought to minimize the impact of cognitive biases on decision-making. Per nominal group technique (24, 25), pre-meeting surveys were used to help panelists engage in creative brainstorming prior to the meeting; having participants describe and justify their ideas in advance was intended to reduce potential for groupthink and facilitator biases (29). We also included an ombuds procedure whereby participants could communicate via a non-research administrator through a direct, private chat. Given the differences in power and perspectives across groups, with more researchers than community interest-holders in attendance, we did not use majority voting procedures to progress ideas forward like many past consensus approaches, although we did ask participants to nominate their top ideas for discussion via pre-meeting surveys and ensured these were discussed each day. We also started each discussion segment with input from peer coaches and/or reflections on participants’ perspectives to center the discussion on community members’ priorities and give community interest-holders power over the meeting contents.

#### Pillar #6: Center diversity of experiences and perspectives, including for “n = 1” experiences.

Although it is common in clinical science to look for generalizable outcomes that will apply to a broad population, our CASCADE approach acknowledges that often, clinical decision-making must consider how research will impact participants in the minority, whether defined by demographic or experiential factors, regardless of sample size. Thus, in addition to adequately powered, pre-registered analyses, we encouraged open discussion of “n = 1” issues, such as challenges that may differentially impact specific subgroups of participants. Here, where robust statistical approaches are not possible, these discussions are anchored with other sources of data – including past research, lived experiences shared by participants or community representatives, and qualitative findings.

#### Pillar #7: Make decisions that are community-relevant, rigorous, and feasible.

A major barrier to the implementation of CBPR in research decision-making, as defined by Israel and colleagues (13), is the task of synthesizing rich, multifaceted patient data into actionable outputs. Prior to translating a hypothesis into action, we required that (1) the action be supported by community, as expressed by peer coaches and/or data collected from participants, (2) the action be supported by at least 2 of the following data sources: quantitative data, qualitative data, past literature, lived experience, (3) the action be feasible within the temporal and financial constraints of the project. Actions that did not meet these criteria were flagged for follow-up outside of the CASCADE panel context, such as to conduct pilot projects or address feasibility barriers through longer-term projects and grants. The panel limited discussion of such endeavors to maximize panel efficiency.

### Procedures

Here, we detail the key chronological sequence of tasks required to plan and execute the CASCADE approach, including how we applied each step within our project-specific CASCADE panel.

Prior to the CASCADE meeting, we articulated our primary goal that was “fixed” within our grant protocol: “Identify how to improve the Project WellCAST algorithm to better match caregivers to feasible, acceptable, and effective supports.” Next, we pre-registered the specific statistical analyses that would be used to guide our panel discussion and report on the project’s OSF.io site, complementing our prior trial registrations. In the context of our panel goal, analyses focused on how the feasibility, acceptability, and efficacy of the clinical trial treatments varied according to participant and treatment characteristics.

Next, we defined our panel structure. We invited all project co-investigators, research staff (including community interest-holders), research assistant trainees, clinical supervisors, clinician trainees, and biostatisticians. Panelists were encouraged to attend as much of the meeting as possible, with the understanding that other commitments may impact attendance; agendas were adjusted to maximize discussion that was relevant to participants with intermittent availability. To maximize participation, we implemented a hybrid model that included in person and remote options for attendance.

Panelists received a pre-meeting survey that requested specific inputs relevant to the planned discussion, with questions designed to parallel key decision points. Prior to the meeting, panelists received several digital items; a subset of documents were also mailed to remote participants. These items included: (1) a preliminary report of findings, with a focus on descriptive data that are used for pre-registered analyses, (2) agenda and slide deck, (3), links to supplemental descriptions of all measures, procedures, and de-identified data for additional use if needed, (4) hand-written thank you note and project “swag” (sticker, pen) to promote a sense of community and belongingness. Digital documents were provided in a secure, password protected cloud folder.

## RESULTS

The Project WellCAST CASCADE panel occurred July 2024 and facilitated by the project PI (BK) who had prior experience leading interdisciplinary groups toward consensus decisions, including through formalized training in agile leadership for moving groups toward action (41); the leader was not a member of the focal participant community (rare disorder caregivers) but experienced some shared identity as a parent.

### Attendees.

Including the facilitator, 27 team members attended across days, including 5 staff who were also community interest-holders, 9 doctoral-level clinical researchers (5 licensed), 3 biostatisticians (two doctoral-level, one masters-level), 8 psychology and special education trainees (2 postdoctoral scholars, 4 graduate students, 2 undergraduate students), 1 research project manager and 1 administrative support staff. Researchers represented 5 institutions across two countries (United States and New Zealand), and community interest-holders represented 4 patient communities.

### Panel Structure.

**Figure 2:**
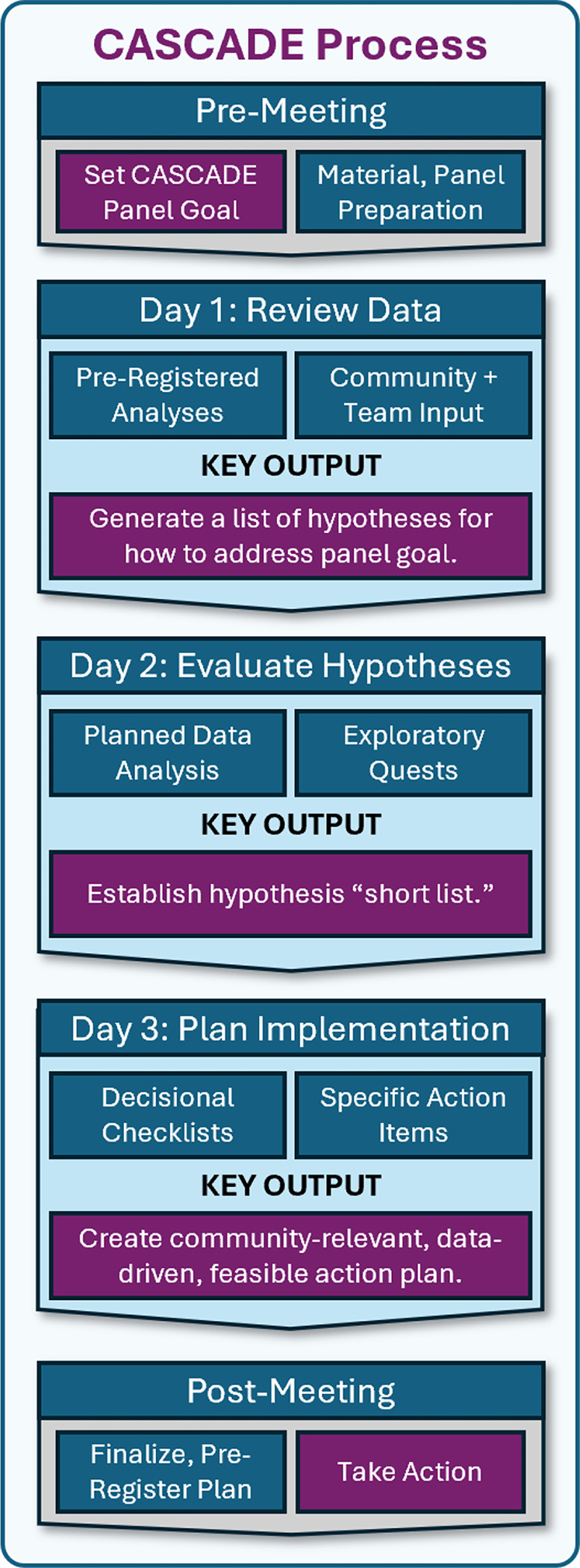
CASCADE three-day itinerary and key outputs

**Figure 3:**
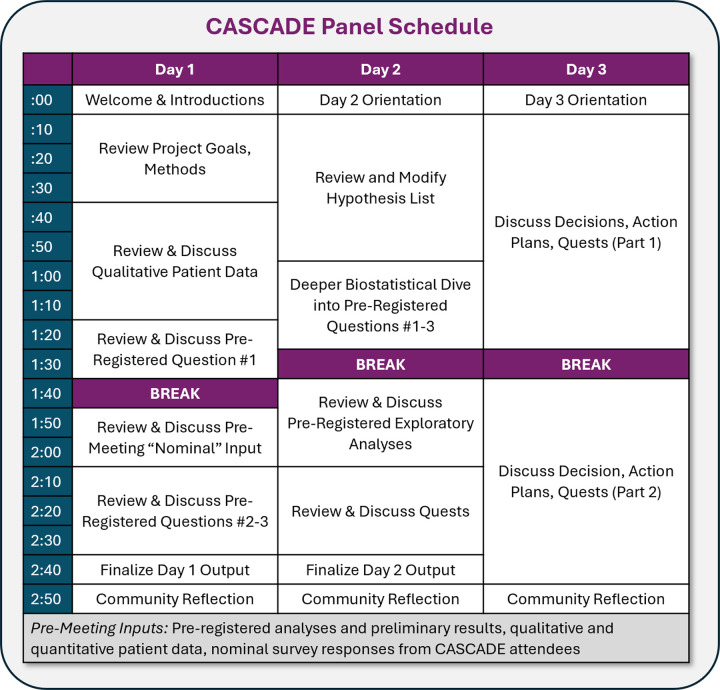
Observed CASCADE Schedule for Project WellCAST Panel (July 2024)

The CASCADE Panel occurred across three consecutive days (Figs. 2 and 3), with sessions held between 2–5PM EST each day to account for multiple time zones of participants, who attended from across the United States and New Zealand. The meeting was administratively supported by an on-site research operations administrator who served as an ombudsperson via secure, private chat and could relay anonymous information to the project team in real time. All attendees were encouraged to update meeting notes in real time via shared documents and were given access to additional technical materials (“Meeting Inputs”) that they could access from personal computers.

### Panel Schedule and Outputs.

Figure 3 details the panel itinerary. Day 1 primarily focused on reviewing project data and generating hypotheses; the end-product was a list of hypotheses about how changes to the algorithm may improve participant outcomes. Day 2 primarily focused on “Quests” through which we conducted exploratory analysis of past WellCAST data and reviewed past literature to estimate the feasibility and impact of various hypothesized improvements; the end-product was a list of final algorithm improvement suggestions. Day 3 focused on establishing a plan for implementing algorithm changes; the end-product was a draft of planned changes that would finalized in the post-panel period by core project staff.

### Hypotheses and Quests.

Across days, 18 specific hypotheses were suggested for consideration, and 12 quests were undertaken to contextualize the relative strengths and weaknesses of these hypotheses. Quests included specific pilot analyses (n = 7; e.g., detailed summaries of why participants dropped out of the study, statistical analyses exploring the degree to which emotional dysregulation related to drop-out), administrative record review (n = 1; e.g., clarifying types of employment in demographic data), and reviews of the literature (n = 5; e.g., surveying the literature for examples of how personality might be related to group treatment dynamics).

### Decisional Outputs.

Of the 18 hypotheses initially suggested for consideration, 12 were candidates for immediate action (feasible) and were discussed for relevance to the community and support from past data. An additional 16 hypotheses were earmarked for later follow-up; for example, although there was enthusiasm to consider how the construct of hope may relate to outcomes, a measure of hope was not in the original dataset, and follow-up discussion was planned to consider adding such a measure. Final justification for each CASCADE-generated decision will be published alongside study findings; in total, 19 decisional changes were selected that aligned with our criteria for community-relevance, empirically supported, and feasible (Pillar #7). Verbal consent from all panelists in attendance was used to determine final consensus.

### Post-Panel Action.

Final edits to the report and the proposed, preregistered algorithm are being sent to the CASCADE team for verification and integration into the next round of project routing decisions, which was scheduled 5 weeks following the CASCADE panel. Following the meeting, project staff re-reviewed all recordings to check for completeness of documentation and verifying all proposed changes met decisional criteria. Given our goal was highly technical in nature (changing an algorithm), there were also several follow-up steps of identifying specific thresholds, updating code, and piloting and debugging updates. Across stages, these technical changes were constrained to the general scope of decisions made during the CASCADE panel, and the final list of changes, including any technical details that were not explicitly discussed during the panel, were sent to all panelists for verification.

## DISCUSSION

Although a variety of decision-making procedures have been developed for medical contexts, existing procedures typically require substantial time and resources and offer minimum opportunity for patient and community input. Here, we describe a new decision-making model, CASCADE (Community-Engaged Approach for Scientific Collaborations and Decisions), designed to systematically integrate scientific and interest-holder inputs to make clinical research decisions. Results from our inaugural CASCADE panel indicated that the methodology facilitated efficient, data-based decision making by a highly interdisciplinary team, with substantial input from community interest-holders. Here, we summarize key takeaways from implementing CASCADE and anticipated next steps for expanding and standardizing this methodology.

A primary takeaway was the efficiency with which decisions were made using the CASCADE approach. Specifically, in less than 9 hours of panel meetings across three consecutive days, our CASCADE team was able to efficiently review data, generate hypotheses, consider the relative strengths and weaknesses of these hypotheses, and make an actionable list of decisions. This efficiency was facilitated by several aspects of our CASCADE approach. First, consistent with the nominal group technique for decision-making (24, 25), a variety of inputs were prepared in advance, including a survey to solicit panelist input. In addition to supporting meeting efficiency, soliciting written input in advance was anticipated to minimize potential cognitive biases (29), consistent with many other consensus-generating models (17, 18). We also leveraged technology to enhance meeting efficiency, including by using AI to rapidly summarize de-identified, non-sensitive meeting inputs. It is important to note that consistent with best practice (40), we did not use AI to code or extract themes from qualitative data, or to analyze any sensitive or identifiable inputs; AI was solely used as a summarizing tool. As AI technology continues to evolve, it will be important to continuously evaluate how to harness the power of AI while also protecting the quality of data and confidentiality of project participants.

A second key takeaway was the high impact of patient community input on panel decisions, which again reflected a variety of intentional strategies in the CASCADE model design. We integrated patient community input directly and indirectly, including by centering input from paid community representatives who were members of the project staff. These team members were highly knowledgeable about project procedures, engaged directly with patients as part of the trial, and could provide highly specific input informed by both their lived experience and project experiences. Anecdotally, many non-community panelists noted that, consistent with the many benefits of CBPR (14), the research team would have likely interpreted results of analyses differently if not for the interest of these team members, who often provided context and nuance that was not possible to detect from numeric data alone. Structurally, we also observed positive outcomes of starting each discussion period with space for community interest-holders to speak first, which ensured that the “seed” for each discussion was centered on the community priorities and needs. Given the compact schedule for our CASCADE panel, starting with community input was critical to maximizing the impact of community-interest holders on project decisions.

Several considerations will motivate future phases of CASCADE model development. First, we will consider who should facilitate CASCADE panels. Here, CASCADE was facilitated by the project PI and developer of the CASCADE model, who had prior formal training in group-based consensus generating procedures. To minimize potential for facilitator-related biases, panel procedures were pre-registered, community interest-holders were called upon first to provide input during each segment, and all decisions were made via consensus. However, these procedures do not fully ameliorate the potential for facilitator bias, and future work should explore potential benefits of objective, external facilitators. Second, community input similarly originated from within the project; this decision reflected the need to protect patient and algorithm information during an ongoing trial. However, future projects could explore creative and secure ways to gather broader community input – such as by preparing a separate pre-panel meeting to discuss broad project questions with patient community representatives and relevant foundations – to improve the scope of community input.

We are also considering several aspects of CASCADE panel structure. First, the specific decision to execute CASCADE across three part-day meetings was somewhat arbitrary, reflecting the estimated minimum time needed to execute activities and the temporal constraints of the clinical trial. Future experimentation could alternate schedules, including those that allow more time to re-solicit panelist input(23) and complete additional quests. Second, we made decisions via consensus, without anonymous voting procedures that are common in decision making models, in part because our community representatives were the minority of panelists. Our panel functioned highly collaboratively and congenially, with no overt conflict across group members or engagement of the ombudsperson that would suggest potential undetected disagreement. Nonetheless, it is possible that panelists did not feel comfortable expressing opinions openly, and procedures such as anonymous votes could be explored. Third, it will be important to consider best-practices for CASCADE outputs, including how recent standardized reporting guidelines for consensus-based decision making(18) could be adapted and optimized for this model. Finally, although our hybrid meeting format facilitated broad, global participation, it is possible that this structure created uneven engagement and feeling of belongingness across members, consistent with past research (42). Future work could consider how best to structure meeting locations, including virtual meeting elements, to maximizing panelist engagement and sense of community

## CONCLUSIONS

The CASCADE model proved to be an efficient and effective model for moving complex inputs toward tangible, actionable decisions in the context of an ongoing clinical trial. Particular strengths of this model included its high efficiency, centering of community interest-holder input, and integration of strategies to reduce cognitive biases inherent to group-based decision making. Next steps will include determining optimal structure for CASCADE panel meetings – including facilitation, timing, format, and pre-meeting inputs. However, at present, the CASCADE model shows promise for supporting rigorous and rapid community-centered decision making, potentially narrowing the current practice gap between best-practice community-integration and consensus-building approaches in medical research.

## Data Availability

Data sharing is not applicable to this article as no human subject datasets were generated or analyzed. Data related to Project WellCAST, as well as project pre-registrations relevant to the methods described in this manuscript, are available at https://osf.io/5j8xn/.

## Abbreviations

AI: Artificial Intelligence
CASCADE: Community-Engaged Approach for Scientific Collaborations and Decisions
Project WellCAST: Supporting the Well-Being of Caregivers via Telehealth

## References

[1] Green LW, Mercer SL. Can Public Health Researchers and Agencies Reconcile the Push from Funding Bodies and the Pull from Committees? Community-Based Participatory Res. 2001;91(12):1926–8. 10.2105/ajph.91.12.1926 PMC1446906 11726367

[2] Harrington RL, Hanna ML, Oehrlein EM, Camp R, Wheeler R, Cooblall C, Defining Patient Engagement in Research: Results of a Systematic Review and Analysis: Report of the ISPOR Patient-Centered Special Interest Group. Value Health. 2020;23(6):677–88. 32540224 10.1016/j.jval.2020.01.019

[3] Sharma AE, Knox M, Mleczko VL, Olayiwola JN. The impact of patient advisors on healthcare outcomes: A systematic review. BMC Health Serv Res. 2017;17(1). 10.1186/s12913-017-2630-4 PMC5651621 29058625

[4] Strauss RP, Sengupta S, Quinn SC, Goeppinger J, Spaulding C, Kegeles SM, The Role of Community Advisory Boards: Involving Communities in the Informed Consent Process. Am J Public Health. 2001;91(12):1938–43. 11726369 10.2105/ajph.91.12.1938 PMC1446908

[5] Graham KL, Green S, Kurlan R, Pelosi JS. A Patient-Led Educational Program on Tourette Syndrome: Impact and Implications for Patient-Centered Medical Education. Teach Learn Med [Internet]. 2014;26(1):34–9. 10.1080/10401334.2013.857339 24405344

[6] Le Cunff AL, Ellis Logan P, Ford R, Martis BL, Mousset I, Sekibo J Co-Design for Participatory Neurodiversity Research: Collaborating With a Community Advisory Board to Design a Research Study. J Particip Res Methods. 2023;4(1).

[7] O’Mara-Eves A, Brunton G, Oliver S, Kavanagh J, Jamal F, Thomas J. The effectiveness of community engagement in public health interventions for disadvantaged groups: A meta-analysis. BMC Public Health. 2015;15(1). 10.1186/s12889-015-1352-y PMC4374501 25885588

[8] Sekhon M, Cartwright M, Francis JJ. Acceptability of healthcare interventions: An overview of reviews and development of a theoretical framework. BMC Health Serv Res. 2017;17(1). 10.1186/s12913-017-2031-8 PMC5267473 28126032

[9] Napier KR, Tones M, Simons C, Heussler H, Hunter AA, Cross M A web-based, patient driven registry for Angelman syndrome: The global Angelman syndrome registry. Orphanet J Rare Dis. 2017;12(1). 10.1186/s13023-017-0686-1 PMC5540301 28764722

[10] Boulanger V, Schlemmer M, Rossov S, Seebald A, Gavin P. Establishing Patient Registries for Rare Diseases: Rationale and Challenges. Pharmaceut Med. 2020;34(3):185–90. 32215853 10.1007/s40290-020-00332-1 PMC7286934

[11] Yates N, Hinkel J. The economics of moonshots: Value in rare disease drug development. Clinical and Translational Science. Volume 15. John Wiley and Sons Inc; 2022. pp. 809–12. 35334152 10.1111/cts.13270 PMC9010265

[12] Lewin K. Action Research and Minority Problems. J Soc Issues. 1946;2(4):34–46.

[13] Israel BA, Schulz AJ, Parker EA, Becker AB, REVIEW OF COMMUNITY-BASED RESEARCH. : Assessing Partnership Approaches to Improve Public Health. 19, Annu Rev Public Health. 1998. 10.1146/annurev.publhealth.19.1.173 9611617

[14] Israel BA, Schultz AJ, Parker EA, Becker AB, Allen AJ, Guzman I R,. Critical Issues in Developing and Following CBPR Principles. In: Community-Based Participatory Research for Health: Advancing Social and Health Equity. 2017. pp. 32–5.

[15] Satcher D. Methods in community-based participatory research for health. Wiley; 2005.

[16] Israel BA, Eng E, Schultz AJ, Parker EA, editors. Methods in Community-Based Participatory Research for Health. San Francisco, CA: Jossey-Bass; 2005.

[17] Bourrée F, Michel P, Salmi LR. Consensus methods: Review of original methods and their main alternatives used in public health. Revue d’Epidemiologie et de Sante Publique. Volume 56. Elsevier Masson SAS; 2008. pp. 415–23. 10.1016/j.respe.2008.09.006 PMC3838316 19013039

[18] Gattrell WT, Logullo P, van Zuuren EJ, Price A, Hughes EL, Blazey P ACCORD (ACcurate COnsensus Reporting Document): A reporting guideline for consensus methods in biomedicine developed via a modified Delphi. PLoS Med. 2024;21(1). 10.1371/journal.pmed.1004326 PMC10805282 38261576

[19] Dalkey NC. The Delphi Method: An Experimental Study of Group Opinion. Santa Monica, CA; 1969.

[20] Nasa P, Jain R, Juneja D. Delphi methodology in healthcare research: How to decide its appropriateness. World J Methodol. 2021;11(4):116–29. 34322364 10.5662/wjm.v11.i4.116 PMC8299905

[21] Avella JR. Delphia Panels: Research Design, Procedures, Advantages, and Challenges. Int J Doctoral Stud. 2016;11:305–21.

[22] Fitch K, Bernstein SJ, Aguilar MD, Burnand B, LaCalle JR, Lazaro P RAND/UCLA appropriateness method user’s manual. Santa Monica, CA; 2000.

[23] Fink A, Kosecoff J, Brook RH. Consensus Methods: Characteristics and Guidelines for Use. 1984. 10.2105/ajph.74.9.979 PMC1651783 6380323

[24] Van de Ven A, Delbecq AL. The Nominal Group as a Research Instrument for Exploratory Health Studies. Am J Public Health. 1972;62(3):338–42. 10.2105/ajph.62.3.337 PMC1530096 5011164

[25] Van De Ven A, Delbeco AL. Nominal versus Interacting Group Processes for Committee Decision-Making Effectiveness [Internet]. Vol. 14, Source: The Academy of Management Journal. 1971. https://about.jstor.org/terms

[26] Manera KE, Johnson DW, Craig JC, Shen JI, Ruiz L, Wang AYM, Patient and caregiver priorities for outcomes in peritoneal dialysis multinational nominal group technique study. Clin J Am Soc Nephrol. 2019;14(1):74–83. 30573659 10.2215/CJN.05380518 PMC6364541

[27] Urquhart-Secord R, Craig JC, Hemmelgarn B, Tam-Tham H, Manns B, Howell M, Patient and Caregiver Priorities for Outcomes in Hemodialysis: An International Nominal Group Technique Study. Am J Kidney Dis. 2016;68(3):444–54. 26968042 10.1053/j.ajkd.2016.02.037

[28] Saposnik G, Redelmeier D, Ruff CC, Tobler PN. Cognitive biases associated with medical decisions: a systematic review. BMC Med Inf Decis Mak. 2016;16(1):1–14. 10.1186/s12911-016-0377-1 PMC5093937 27809908

[29] Bhandari S, Hallowell MR. Identifying and Controlling Biases in Expert-Opinion Research: Guidelines for Variations of Delphi, Nominal Group Technique, and Focus Groups. 2021.

[30] Ross L, Greene D, House P. The False Consensus Effect: An Egocentric Bias in Social Perception and Attribution Processes. J Exp Soc Psychol. 1976;279–301.

[31] Adelman C. Kurt Lewin and the Origins of Action Research. Educ Action Res [Internet]. 1993;1(1):7–4. 10.1080/0965079930010102

[32] Minkler M, Wallerstein N. Introduction to community-based participatory research: New issues and emphases. In: Community-based particpatory research for health: From process to outcomes. 2008. pp. 5–23.

[33] Bowker L. Interdisciplinary Research Methods: Considering the Potential of Community-based Participatory Research in Translation. J Translation Stud. 2021;1(1):13–26.

[34] Doran GT. There’s a SMART Way to Write Management’s Goals and Objectives. J Manage Rev. 1981;70:35–6.

[35] OpenAI. ChatGPT.

[36] Nosek BA, Ebersole CR, DeHaven AC, Mellor DT. The preregistration revolution. Proc Natl Acad Sci U S A. 2018;115(11):2600–6. 29531091 10.1073/pnas.1708274114 PMC5856500

[37] Dickersin K, Rennie MD. Registering Clinical Trials [Internet]. Available from: www.jama.com. 10.1001/jama.290.4.516 12876095

[38] Simmons JP, Nelson LD, Simonsohn U. False-positive psychology: undisclosed flexibility in data collection and analysis allows presenting anything as significant. Psychol Sci. 2011;22(11):1359–66. 22006061 10.1177/0956797611417632

[39] Google. Google Docs.

[40] Morgan DL. Exploring the Use of Artificial Intelligence for Qualitative Data Analysis: The Case of ChatGPT. Int J Qual Methods. 2023;22.

[41] Morrison E, Hutcheson S, Nilsen E, Fadden J, Franklin N. Strategic doing: Ten skills for agile leadership. Wiley; 2019.

[42] Sacco DF, Ismail MM. Social belongingness satisfaction as a function of interaction medium: Face-to-face interactions facilitate greater social belonging and interaction enjoyment compared to instant messaging. Comput Hum Behav. 2014;36:359–64.

